# Supplementary material for: Rapid turnover of life-cycle-related genes in the brown algae
Source: Genome Biol. 2019 Feb 14;20:35. doi: 10.1186/s13059-019-1630-6 (PMC6374913; doi:10.1186/s13059-019-1630-6)
Supplement: Supplementary file 3 — Protein alignments, corresponding Gblocks html file and CDS sequences for the lineage conserved gametophyte-biased genes. (ZIP 2109 kb) [file 13059_2019_1630_MOESM3_ESM.zip › Con_GA_alignments/Ec-16_002110.aa_ali.fasta-gb.htm]

Ec-16\_002110.aa.fasta.trans.aa\_ali.fasta


## Gblocks 0.91b Results

Processed file: **Ec-16\_002110.aa.fasta.trans.aa\_ali.fasta**  
Number of sequences: **2**  
Alignment assumed to be: **Protein**  
New number of positions: **417** (selected positions are underlined in blue)

```
                         10        20        30        40        50        60
                 =========+=========+=========+=========+=========+=========+
Ec-16_002110     MFRDPTEPGSG------------TRRFSSAVYSSRLAGATRTFRDP------------II
MSTRG.42706.1    MARKPRENASGSTAIATEAASRRQKRLSLTAYNNRLSGATRTFRDPFEVDVDVRRRFSTT
                 ###########              #####################              


                         70        80        90       100       110       120
                 =========+=========+=========+=========+=========+=========+
Ec-16_002110     SHGLETAGTAGGRSSTG-------------RRGGSRSDM-------DRMIPEG--ASDSS
MSTRG.42706.1    GHSSSSSTMAGGTRAARHPSLVVGMPWSAIRRSRSPSFVDSGSGITSRMLATTTEVARES
                  ###########                                               #


                        130       140       150       160       170       180
                 =========+=========+=========+=========+=========+=========+
Ec-16_002110     PATTAGGNQTSTANSSSRRASLFEWLVAKKGTGGGGEFSQYMSAVECGSDEQQDAVHNEE
MSTRG.42706.1    ATTAAVERRASAPTRAGRRVSIQEWWAAKVGVAVK---SGTQTSQSLRGSEREGFLEDEY
                 ###############################                   ##########


                        190       200       210       220       230       240
                 =========+=========+=========+=========+=========+=========+
Ec-16_002110     QDFGRGFSDYESSMDMNSLRRQAAVVEVLAEEGATTTIPSIDNISSDPPLTAEALEHHTR
MSTRG.42706.1    DNTDRPRPGDESDYDEAS---QSAMADVPTRENMIEAEVDDKEIAS--PASSIDINDPCA
                 ##################   ###########                            


                        250       260       270       280       290       300
                 =========+=========+=========+=========+=========+=========+
Ec-16_002110     THSSGKSNRSEGSASAALNGNDTKHILAASAGRSLPRPRSTSKSSHTGSDDEAGDAVLPE
MSTRG.42706.1    EASRDFRTYLEG----EYDSGSGRSTDVSGDGEVGMSPLSTLFRRISRGDDEGGDPVPTT
                   ##########                   #############################


                        310       320       330       340       350       360
                 =========+=========+=========+=========+=========+=========+
Ec-16_002110     FHQSHD-EAQEELTIDEPTTPAAVDINDIYADDVTGGESRRVTFLSGTMGLTVDISDPMQ
MSTRG.42706.1    RQELHQTNGKEETAFDRP----AAVVKDIYSNTSEHGEPRRVAFLSGTLGLTVDTTVPIY
                 #####                 ######################################


                        370       380       390       400       410       420
                 =========+=========+=========+=========+=========+=========+
Ec-16_002110     PQPSDE----FDREHRQQHTKHDDYDQWPASPCSGVILDTSPRYGTLQAFRKEMASRGIN
MSTRG.42706.1    PHPSDEGHWKEHSQHQQQQRTHADREGWPASPCSGVILETSPRYGSLQAFRKEMSSRGID
                 ######        ##############################################


                        430       440       450       460       470       480
                 =========+=========+=========+=========+=========+=========+
Ec-16_002110     AQMFHTGWKGSFLPPRDIRLFMTDSRKASVLYWRYKTGPFGRGAAAGANQGLGAEDRRVI
MSTRG.42706.1    AQMFHTGWKARFLPPRDIRLFLTDSRKSSVLFWRYKKKSRGGRRGTAATGDLGPEDRRVI
                 ############################################################


                        490       500       510       520       530       540
                 =========+=========+=========+=========+=========+=========+
Ec-16_002110     PLSSLANIGPAELYRQGPPSS----RRNLLSWLSIPSSLRRASEDAPVGYGKIQKDKDLD
MSTRG.42706.1    PLSSMANIGPAEVYRRSASATRGRSSRRRSSWVPIGAPLRWGAEESAYSRGDSQ-GKDFD
                 ###############           ############################      


                        550       560       570       580       590       600
                 =========+=========+=========+=========+=========+=========+
Ec-16_002110     SVDKG---TAGLVEAVPVLALSYWVEGEPRWVGDQPKRLLVRPDSLDSFQRLSHGLRLCC
MSTRG.42706.1    SCEKDETSALGPEEDVPVVALSYWGNGEPRWEGDKPKRLLVKPESFDTFQRLTNGLRLCC
                           ##################################################


                        610
                 =========+======
Ec-16_002110     LHFHNYSCPQDLEDIR
MSTRG.42706.1    LHFYNHSCPQDLEDIR
                 ################
```

```
Parameters used
Minimum Number Of Sequences For A Conserved Position: 2
Minimum Number Of Sequences For A Flanking Position: 2
Maximum Number Of Contiguous Nonconserved Positions: 8
Minimum Length Of A Block: 10
Allowed Gap Positions: None
Use Similarity Matrices: Yes
```

```
Flank positions of the 12 selected block(s)
Flanks: [1  11]  [26  46]  [62  72]  [120  151]  [171  198]  [202  212]  [243  252]  [272  305]  [323  366]  [375  495]  [507  534]  [551  616]  

New number of positions in Ec-16_002110.aa.fasta.trans.aa_ali.fasta-gb:  417  (67% of the original 616 positions)
```
